# Dataset on the concentrations of anticoagulant rodenticides in raptors from the Canary Islands with geographic information

**DOI:** 10.1016/j.dib.2021.106744

**Published:** 2021-01-13

**Authors:** Cristian Rial-Berriel, Andrea Acosta-Dacal, Miguel Ángel Cabrera Pérez, Alejandro Suárez-Pérez, Ayose Melián Melián, Manuel Zumbado, Luis Alberto Henríquez Hernández, Norberto Ruiz-Suárez, Ángel Rodríguez Hernández, Luis D. Boada, Ana Macías Montes, Octavio P. Luzardo

**Affiliations:** a Toxicology Unit, Research Institute of Biomedical and Health Sciences (IUIBS), University of Las Palmas de Gran Canaria, Paseo Blas Cabrera s/n, 35016 Las Palmas de Gran Canaria, Spain; b General Directorate to Combat Climate Change and the Environment, Biodiversity Service, Canary Islands Government, Plaza de los Derechos Humanos, 22, 35071 Las Palmas de Gran Canaria, Spain; c “Tafira” Wildlife Recovery Center. Ctra. Del Centro, 35017 Las Palmas de Gran Canaria, Spain; d Gestión y Planeamiento Territorial y Medioambiental, S.A. (GESPLAN), Canary Islands Government, C / León y Castillo 54, bajo, 35003 Las Palmas de Gran Canaria, Spain; e Spanish Biomedical Research Center in Physiopathology of Obesity and Nutrition (CIBERObn), Spain; f Department of Nutrition, Genetics and Ethology, Faculty of Veterinary Medicine, Ghent University, Heidestraat 19, 9820 Merelbeke, Belgium

**Keywords:** Geographic information system, Long-eared owl, Common kestrel, Common buzzard, Egyptian vulture, SGAR

## Abstract

The dataset presented in this article supports “Intensive livestock farming as a major determinant of the exposure to anticoagulant rodenticides in raptors of the Canary Islands (Spain)” (Rial-Berriel et al., 2020). A Geographic Information System (GIS) analysis on the influence of the influence of livestock activity on exposure to anticoagulant rodenticides in raptors in the Canary Islands was performed. This dataset provides geographic information on the localization of each raptor (either positive or negative for anticoagulant rodenticides, *n* = 308), as well as the concentrations of each compound found in their livers. In addition, we present complementary analyses to those included in the main article, such as the detailed analysis of the farming activity influence on anticoagulant rodenticide exposure of raptors, by island and by raptor species.

## Specifications Table

**Table:** 

Subject	Environmental Chemistry
Specific subject area	Census of raptor specimens for contaminant biomonitoring
Type of data	Figures (processed data), and the corresponding raw data (table)
How data were acquired	Ultra-high performance liquid chromatography coupled to triple quadrupole mass spectrometry (LC-MS/MS) GPS devices
Data format	Raw and analysed
Parameters for data collection	The data were systematically collected in the course of forensic investigations of incidents affecting wildlife. The main conditions for starting the data collection were that: • The animal was a raptor bird nesting in the Canary Islands, • Found dead or died/euthanized within a week of its admission to a wildlife recovery center. • With fresh liver tissue available, • With georeferenced data of the location where the carcass or severely affected animal was found.
Description of data collection	All the available information about the animal (species and subspecies, age, sex, the possible cause of death, etc. . .), as well as the coordinates of the place where the animal/cadaver was found, collected with a centimetre precision GPS device by the environmental officers, were obtained from the reports of sample collection and the medical histories opened in the wildlife recovery centres of the Canary Islands. The quantification data of anticoagulant rodenticides in raptor were obtained analysing a series of 308 livers from January 2011 to May 2020.
Data source location	Institution: Toxicology Unit, Clinical Sciences Department, Universidad de Las Palmas de Gran Canaria City/Town/Region: Las Palmas de Gran Canaria (Gran Canaria, Canary Islands) Country: Spain
Data accessibility	With the article
Related research article	Intensive livestock farming as a major determinant of the exposure to anticoagulant rodenticides in raptors of the Canary Islands (Spain) https://doi.org/10.1016/j.scitotenv.2020.144386

## Value of the Data

• The data that we present in this article are very useful to researchers who carry out biomonitoring of pesticides. They will also contribute in a very relevant way to the elaboration of the map of the exposure to rodenticides of raptors in Europe (LIFE APEX projects, European Raptor Biomonitoring Facility), as well as to implement the Information Platform for Chemical Monitoring (IPCheM) database, which is the European Commission's reference access point for searching, accessing and retrieving chemical occurrence data collected and managed in Europe.
• By providing not only pollution data, but also the GPS coordinates of 308 raptor specimens, some of them belonging to species very rarely observed in Europe (the case of Eleanora's falcon and Barbary falcon), allows feeding databases, such as the Census of raptor specimens for pan-European contaminant biomonitoring.
• In addition, the censuses that are currently being carried out in Europe focus in a special way on those species that have a pan-European distribution, since the contamination data in raptors is crossed with those of the human population and in environmental samples of each country. In this work, we provide data from numerous specimens of the species that have been identified as being of greatest interest for this purpose: *Buteo buteo* (*n* = 53); *Falco tinnunculus* (*n* = 83); *Tyto alba* (*n* = 8); *Asio otus* (*n* = 68); as well as data from many other species of interest.

## 1 Data Description

The data presented here are part of a larger series of 831 animals that have been investigated for anticoagulant rodenticides in the Canary Islands over a decade (2011–2020), and which are presented in the article by Rial-Berriel et al. [1]. The data detailed at the individual level here focus on the 308 raptors included in that series. We have decided to include all the raptor specimens investigated, both those positive for anticoagulants and those negative, due to the importance of having georeferenced data of the specimens suitable for future analysis of contaminants at a pan-European level. Table 1 contains the details of all the individuals investigated, including species and subspecies, geographical data of their location (island, municipality, and GPS coordinates), and the individual concentrations of the 5 anticoagulant rodenticides detected in the series (brodifacoum, bromadiolone, difenacoum, difethialone, and flocoumafen). The shortest distance from the location of the bird of prey to the nearest cattle farm is also presented and including the type of livestock farmed.

**Table 1:** Identification of each bird included in the study, with detailed information about the location where the corpse was found, including GPS coordinates, and about the concentration of anticoagulant rodenticides detected (replicate analysis are provided).


																SUM OF RODENTICIDES			
RAPTOR						BRODIFACOUM	BRODIFACOUM	BROMADIOLONE	BROMADIOLONE	DIFENACOUM	DIFENACOUM	DIFETHIALONE	DIFETHIALONE	FLOCOUMAFEN	FLOCOUMAFEN	(using averaged	Nº	Distance to the	Type of
SPECIES	YEAR	ISLAND	MUNICIPALITY	UTMX	UTMY	measurement 1	measurement 2	measurement 1	measurement 2	measurement 1	measurement 2	measurement 1	measurement 2	measurement 1	measurement 2	measurements)	RODENTICIDES	nearest farm (m)	livestock
*Accipiter nisus granti*	2020	Gran Canaria	Moya	454582	3104241	34.32	26.96	2.71	2.93	0.72	0.90			0.20	0.24	34.49	4	315	Goat/Sheep
*Accipiter nisus granti*	2020	Gran Canaria	Telde	458011	3096342	12.54	9.86	0.19	0.21	0.36	0.44					11.8	3	940	Cattle
*Accipiter nisus granti*	2019	Tenerife	Güimar	359845	3135597	448.00	352.00									400	1	1420	Cattle
*Accipiter nisus granti*	2019	Tenerife	Güimar	359845	3135597											0	0	1420	Cattle
*Accipiter nisus granti*	2019	Tenerife	Güimar	359845	3135597											0	0	1420	Cattle
*Accipiter nisus granti*	2020	Gran Canaria	Las Palmas de Gran Canaria	454183	3107904	2.45	2.87	0.22	0.24							2.89	2	1432	Cattle
*Accipiter nisus granti*	2019	Gran Canaria	Telde	459098	3096981	5.34	6.26									5.8	1	2040	Goat/Sheep
*Accipiter nisus granti*	2014	Gran Canaria	Moya	441131	3107838											0	0	2580	Goat/Sheep
*Accipiter nisus granti*	2016	Gran Canaria	Artenara	432827	3098128											0	0	3722	Goat/Sheep
*Actitis hypoleucos*	2015	Fuerteventura	Antigua	610682	3140741											0	0	7585	Pork
*Asio otus canariensis*	2020	Gran Canaria	Agüimes	452323	3084882	31.07	36.47			2.90	3.62	28.03	30.37	0.80	1.00	67.13	4	150	Goat/Sheep
*Asio otus canariensis*	2020	Gran Canaria	La Aldea de San Nicolás	447360	3104609	64.18	75.34	5.70	6.18	3.38	4.22					79.5	3	215	Pork
*Asio otus canariensis*	2018	La Gomera	San Sebastian de la Gomera	293178	3110572	20.56	25.64	37.15	40.25							61.8	2	277	Pork
*Asio otus canariensis*	2019	Gran Canaria	San Bartolomé de Tirajana	441414	3073783	34.44	42.96	2.02	2.18							40.8	2	348	Goat/Sheep
*Asio otus canariensis*	2019	Gran Canaria	Santa María de Guía	437346	3107889	21.18	26.42	3.65	3.95	3.56	4.44			0.45	0.56	32.1	4	420	Cattle
*Asio otus canariensis*	2020	Gran Canaria	San Mateo	445964	3099016	1.87	2.33	41.98	45.48							45.83	2	425	Pork
*Asio otus canariensis*	2020	Gran Canaria	Moya	444454	3110673	207.46	258.74	11.90	12.90					0.30	0.38	245.84	3	500	Goat/Sheep
*Asio otus canariensis*	2019	La Gomera	Vallehermoso	277429	3117336	8.10	10.10	15.17	16.43							24.9	2	504	Pork
*Asio otus canariensis*	2019	La Gomera	Alajeró	279237	3108868	26.21	20.59	34.37	37.23							59.2	2	504	Pork
*Asio otus canariensis*	2020	Gran Canaria	Agüimes	457380	3085646	8.40	6.60			1.25	1.55					8.9	2	520	Pork
*Asio otus canariensis*	2019	La Gomera	San Sebastian de la Gomera	291600	3110450	71.34	56.06									63.7	1	520	Pork
*Asio otus canariensis*	2020	Gran Canaria	Arucas	448896	3111130	44.12	34.66									39.39	1	523	Pork
*Asio otus canariensis*	2020	Gran Canaria	Las Palmas de Gran Canaria	457274	3101765	142.96	112.32	0.82	0.70					0.59	0.73	129.06	3	540	Pork
*Asio otus canariensis*	2018	Tenerife	Icod de los Vinos	332484	3137697	1.91	2.39	4.75	4.05	1.07	1.33					7.75	3	558	Cattle
*Asio otus canariensis*	2018	Tenerife	Garachico	328159	3138215	13.84	17.26			2.72	3.40					18.61	2	595	Cattle
*Asio otus canariensis*	2016	Gran Canaria	Santa Brígida	455403	3100630			9.85	8.39							9.12	1	599	Goat/Sheep
*Asio otus canariensis*	2019	Gran Canaria	Arucas	449698	3111641	11.04	13.76									12.4	1	611	Goat/Sheep
*Asio otus canariensis*	2019	Tenerife	Garachico	327088	3138866	39.34	49.06			13.08	16.32					58.9	2	615	Cattle
*Asio otus canariensis*	2019	Tenerife	Garachico	327088	3138866											0	0	615	Cattle
*Asio otus canariensis*	2019	Tenerife	Garachico	327088	3138866											0	0	615	Cattle
*Asio otus canariensis*	2019	Tenerife	Icod de los Vinos	333648	3138294	12.55	15.65	86.94	74.06	2.58	3.22					97.5	3	630	Cattle
*Asio otus canariensis*	2018	Tenerife	La Orotava	349264	3139111	21.46	26.76	15.66	13.34					2.35	2.93	41.25	3	700	Pork
*Asio otus canariensis*	2017	Gran Canaria	Santa Brígida	445895	3099567											0	0	700	Pork
*Asio otus canariensis*	2017	Gran Canaria	Santa Brígida	445379	3099148	20.92	26.09	42.01	35.79							62.4	2	700	Pork
*Asio otus canariensis*	2020	Gran Canaria	Las Palmas de Gran Canaria	453517	3107995	8.23	9.67									8.95	1	708	Goat/Sheep
*Asio otus canariensis*	2019	Gran Canaria	Arucas	448362	3112107	47.05	55.23	1.62	1.38					1.25	1.55	54.04	3	717	Goat/Sheep
*Asio otus canariensis*	2019	Tenerife	Garachico	327929	3139211	9.14	10.74			3.45	3.67					13.5	2	740	Cattle
*Asio otus canariensis*	2017	Gran Canaria	Las Palmas de Gran Canaria	456453	3101685	111.81	131.25									121.53	1	846	Pork
*Asio otus canariensis*	2020	Gran Canaria	Galdar	437782	3109413	156.22	183.38	1.36	1.44			0.48	0.52	4.66	4.94	176.5	4	890	Cattle
*Asio otus canariensis*	2019	Gran Canaria	Telde	457693	3096377	16.04	18.84			4.95	5.25					22.54	2	898	Cattle
*Asio otus canariensis*	2020	Gran Canaria	Moya	444415	3109696	32.57	38.23	2.94	3.12	1.09	1.15	0.27	0.29			39.83	4	932	Pork
*Asio otus canariensis*	2020	Gran Canaria	Moya	443379	3110260	9.53	11.19									10.36	1	980	Goat/Sheep
*Asio otus canariensis*	2020	Gran Canaria	Santa María de Guía	438138	3110107	27.49	32.27	9.17	9.73	0.65	0.69					40	3	1104	Goat/Sheep
*Asio otus canariensis*	2016	Gran Canaria	Las Palmas de Gran Canaria	454870	3103678	136.75	160.53	65.42	69.46	2.70	2.86					218.86	3	1115	Goat/Sheep
*Asio otus canariensis*	2016	Gran Canaria	Las Palmas de Gran Canaria	454870	3103678	67.34	79.06									73.2	1	1115	Goat/Sheep
*Asio otus canariensis*	2020	Gran Canaria	Valsequillo	4513691	3095938											0	0	1140	Goat/Sheep
*Asio otus canariensis*	2019	Gran Canaria	Arucas	449842	3112531	5.04	3.96	4.46	4.74							9.1	2	1148	Goat/Sheep
*Asio otus canariensis*	2016	La Gomera	Vallehermoso	277601	3115588	149.18	117.22	22.41	23.79							156.3	2	1384	Pork
*Asio otus canariensis*	2019	La Gomera	Vallehermoso	276852	3117893	37.74	29.66			3.69	3.91					37.5	2	1395	Pork
*Asio otus canariensis*	2016	Gran Canaria	Tejeda	441406	3097942	264.19	207.57			71.23	75.63					309.31	2	1449	Cattle
*Asio otus canariensis*	2017	Gran Canaria	San Mateo	444207	3096578	11.91	9.35	32.64	34.66	3.72	3.96					48.12	3	1603	Cattle
*Asio otus canariensis*	2016	La Gomera	Alajeró	279309	3110804			10.89	11.57							11.23	1	1742	Pork
*Asio otus canariensis*	2018	Tenerife	Icod de los Vinos	331062	3137692	61.60	48.40									55	1	1786	Cattle
*Asio otus canariensis*	2018	Tenerife	Icod de los Vinos	334580	3140044	12.56	9.86	3.98	4.22							15.31	2	1955	Cattle
*Asio otus canariensis*	2019	Gran Canaria	Telde	458537	3096938											0	0	1970	Goat/Sheep
*Asio otus canariensis*	2017	Tenerife	La Orotava	349544	3140835	16.86	13.24									15.05	1	2008	Cattle
*Asio otus canariensis*	2017	Tenerife	La Orotava	349601	3140971			20.56	25.64							23.1	1	2008	Cattle
*Asio otus canariensis*	2017	Gran Canaria	San Mateo	445037	3093464											0	0	2250	Cattle
*Asio otus canariensis*	2018	Tenerife	Icod de los Vinos	332418	3140319	20.05	23.53			14.16	15.04					36.39	2	2314	Cattle
*Asio otus canariensis*	2020	Gran Canaria	Arucas	448585	3107247											0	0	2430	Goat/Sheep
*Asio otus canariensis*	2017	Tenerife	Icod de los Vinos	333662	3140549	220.80	259.20									240	1	2573	Cattle
*Asio otus canariensis*	2011	Gran Canaria	Santa María de Guía	437802	3114283	68.89	80.87	456.37	569.17	10.28	10.92					598.25	3	2794	Goat/Sheep
*Asio otus canariensis*	2017	Tenerife	El Tanque	325534	3135980			5.87	7.33							6.6	1	3046	Cattle
*Asio otus canariensis*	2019	Gran Canaria	Telde	461042	3099842											0	0	3050	Goat/Sheep
*Asio otus canariensis*	2018	La Gomera	Vallehermoso	274597	3117378											0	0	3270	Pork
*Asio otus canariensis*	2020	Tenerife	Icod de los Vinos	331220	3135855											0	0	3286	Goat/sheep
*Asio otus canariensis*	2018	Tenerife	Icod de los Vinos	335500	3140260			2.79	3.47							3.13	1	3430	Cattle
*Asio otus canariensis*	2017	Tenerife	El Tanque	325537	3135796											0	0	3567	Cattle
*Asio otus canariensis*	2017	Tenerife	Santiago del Teide	322325	3126701											0	0	3620	Goat/Sheep
*Asio otus canariensis*	2016	Gran Canaria	San Mateo	441262	3098794											0	0	4095	Pork
*Asio otus canariensis*	2016	Gran Canaria	San Mateo	443791	3095962											0	0	4181	Pork
*Asio otus canariensis*	2020	Tenerife	Santa Cruz de Tenerife	380799	3156456											0	0	4556	Goat/sheep
*Asio otus canariensis*	2017	Tenerife	El Tanque	352118	3134946											0	0	4566	Pork
*Asio otus canariensis*	2020	Lanzarote	Teguise	642186	3212326											0	0	4788	Goat/sheep
*Asio otus canariensis*	2017	Tenerife	El Tanque	324844	3134546	6.32	7.88									7.1	1	5055	Cattle
*Asio otus canariensis*	2018	La Gomera	San Sebastian de la Gomera	284046	3116134	23.16	28.88									26.02	1	6260	Pork
*Asio otus canariensis*	2018	La Gomera	San Sebastian de la Gomera	284356	3116998					1.34	1.14					1.24	1	6260	Pork
*Asio otus canariensis*	2019	Fuerteventura	Pájara	587386	3136159	2.55	3.19	0.74	0.92							3.7	2	7889	Goat/Sheep
*Buteo buteo insularum*	2020	Gran Canaria	Ingenio	456965	3089566	7.92	9.88			1.84	1.56					10.6	2	310	Goat/Sheep
*Buteo buteo insularum*	2015	Gran Canaria	Valsequillo	449544	3094810	709.01	884.27	109.83	116.63	51.72	44.06					957.76	3	365	Pork
*Buteo buteo insularum*	2016	Gran Canaria	Valleseco	441656	3103060	19.14	23.87	31.72	33.68							54.2	2	400	Pork
*Buteo buteo insularum*	2014	Gran Canaria	Teror	445691	3102357											0	0	580	Goat/Sheep
*Buteo buteo insularum*	2014	Gran Canaria	Teror	445691	3102357	19.09	23.81	34.60	36.74							57.12	2	580	Goat/Sheep
*Buteo buteo insularum*	2018	La Gomera	Vallehermoso	278333	3116479	34.89	43.51									39.2	1	625	Pork
*Buteo buteo insularum*	2019	Gran Canaria	San Mateo	446859	3098882	2.34	2.92	5.78	6.14							8.59	2	735	Pork
*Buteo buteo insularum*	2020	Gran Canaria	Arucas	447622	3113963	68.13	84.97	149.59	158.85	25.27	21.53					254.17	3	786	Pork
*Buteo buteo insularum*	2017	Fuerteventura	Antigua	602696	3139347	4.68	5.84	1.47	1.57							6.78	2	910	Pork
*Buteo buteo insularum*	2016	Gran Canaria	Valleseco	446323	3100270	340.67	267.67	181.97	193.23							491.77	2	919	Pork
*Buteo buteo insularum*	2016	Gran Canaria	Valleseco	446323	3100270											0	0	919	Pork
*Buteo buteo insularum*	2018	Gran Canaria	Arucas	450882	3109182									123.87	131.53	127.7	1	922	Goat/Sheep
*Buteo buteo insularum*	2018	La Palma	Mazo	228158	3162869			22.70	24.10							23.4	1	967	Cattle
*Buteo buteo insularum*	2018	La Palma	Mazo	228256	3162049			119.12	126.48							122.8	1	967	Cattle
*Buteo buteo insularum*	2020	Gran Canaria	Galdar	437673	3108975	134.89	105.99			3.46	2.94					123.64	2	1140	Pork
*Buteo buteo insularum*	2019	Gran Canaria	Las Palmas de Gran Canaria	456551	3106437	2.91	2.29	1.62	1.38							4.1	2	1150	Pork
*Buteo buteo insularum*	2014	Gran Canaria	Artenara	438018	3100502											0	0	1196	Goat/Sheep
*Buteo buteo insularum*	2020	Gran Canaria	San Mateo	444505	3099749	29.06	22.84	19.68	16.76	7.94	6.76					51.52	3	1234	Pork
*Buteo buteo insularum*	2017	La Palma	El Sauzal	362492	3146442	112.00	88.00	36.99	31.51	18.66	15.90					151.53	3	1279	Cattle
*Buteo buteo insularum*	2018	Tenerife	La Matanza de Acentejo	358634	3146709	16.89	13.27	132.95	113.25							138.18	2	1310	Pork
*Buteo buteo insularum*	2019	Gran Canaria	Telde	457380	3096726											0	0	1330	Goat/Sheep
*Buteo buteo insularum*	2015	Gran Canaria	Telde	454049	3097857			25.54	21.76							23.65	1	1375	Cattle
*Buteo buteo insularum*	2018	Tenerife	El Tanque	326925	3137290	6.23	4.89	16.96	14.44	8.50	7.24					29.13	3	1413	Cattle
*Buteo buteo insularum*	2013	Gran Canaria	Galdar	439904	3102452											0	0	1468	Pork
*Buteo buteo insularum*	2013	Gran Canaria	Galdar	439904	3102452	17.55	13.79	35.77	30.47							48.79	2	1468	Pork
*Buteo buteo insularum*	2011	Gran Canaria	San Mateo	445573	3099452			23.38	19.92							21.65	1	1469	Pork
*Buteo buteo insularum*	2017	Tenerife	La Victoria de Acentejo	357709	3144010	26.70	33.30	59.40	50.60							85	2	1493	Pork
*Buteo buteo insularum*	2019	Gran Canaria	San Bartolomé de Tirajana	446430	3086617	3.34	4.16									3.75	1	1503	Pork
*Buteo buteo insularum*	2017	Gran Canaria	San Mateo	444425	3093490	12.02	14.99	131.33	111.87							135.1	2	1705	Cattle
*Buteo buteo insularum*	2017	Gran Canaria	San Mateo	444425	3093490	9.97	12.43	96.55	82.25	34.67	29.53					132.7	3	1705	Cattle
*Buteo buteo insularum*	2014	Gran Canaria	Arucas	447168	3111519	56.03	69.89	47.69	40.63	25.11	21.39					130.37	3	1800	Goat/Sheep
*Buteo buteo insularum*	2014	Gran Canaria	Arucas	447168	3111519											0	0	1800	Goat/Sheep
*Buteo buteo insularum*	2018	Tenerife	Buenavista del Norte	315335	3138528											0	0	1871	Pork
*Buteo buteo insularum*	2017	Gran Canaria	Valleseco	443073	3099523											0	0	2126	Pork
*Buteo buteo insularum*	2017	Gran Canaria	Valleseco	443073	3099523	29.01	36.19	80.24	68.36	11.62	12.58					119	3	2126	Pork
*Buteo buteo insularum*	2011	Gran Canaria	Tejeda	441863	3100156											0	0	2342	Pork
*Buteo buteo insularum*	2011	Gran Canaria	Tejeda	441863	3100156	8.65	10.79	11.84	12.82							22.05	2	2342	Pork
*Buteo buteo insularum*	2018	Tenerife	Buenavista del Norte	317195	3139973											0	0	2627	Pork
*Buteo buteo insularum*	2018	Tenerife	Buenavista del Norte	315602	3139247	9.46	11.80	105.79	114.61							120.83	2	2648	Pork
*Buteo buteo insularum*	2018	Tenerife	Buenavista del Norte	315913	3139006					9.41	10.19					9.8	1	2648	Pork
*Buteo buteo insularum*	2011	Gran Canaria	Tejeda	441807	3093073	12.57	15.67	30.84	33.40							46.24	2	2859	Pork
*Buteo buteo insularum*	2011	Gran Canaria	Tejeda	441807	3093073			1.27	1.37							1.32	1	2859	Pork
*Buteo buteo insularum*	2017	Gran Canaria	San Bartolomé de Tirajana	439141	3090223	2.23	2.78									2.5	1	3280	Pork
*Buteo buteo insularum*	2018	Tenerife	Buenavista del Norte	319935	3135622											0	0	3848	Pork
*Buteo buteo insularum*	2011	Gran Canaria	Artenara	436337	3097463											0	0	3960	Goat/Sheep
*Buteo buteo insularum*	2018	Fuerteventura	Puerto del Rosario	609021	3152412											0	0	4052	Pork
*Buteo buteo insularum*	2020	Tenerife	Guía de Isora	327433	3122925											0	0	4322	Goat/sheep
*Buteo buteo insularum*	2013	Gran Canaria	Artenara	426275	3101266											0	0	5249	Goat/Sheep
*Buteo buteo insularum*	2020	Lanzarote	Haría	617377	3211234											0	0	6322	Goat/sheep
*Buteo buteo insularum*	2020	Gran Canaria	Mogan	428829	3083419											0	0	6345	Goat/sheep
*Buteo buteo insularum*	2018	La Gomera	Hemigua	284932	3115997											0	0	6350	Pork
*Buteo buteo insularum*	2014	Gran Canaria	Tejeda	434124	3091498											0	0	8395	Pork
*Buteo buteo insularum*	2019	Gran Canaria	San Bartolomé de Tirajana	431763	3078873											0	0	12700	Pork
*Circus aeruginosus*	2019	Gran Canaria	Las Palmas de Gran Canaria	457499	3111772											0	0	4015	Pork
*Falco eleonorae*	2014	Gran Canaria	Tejeda	440918	3098515											0	0	4220	Goat/Sheep
*Falco eleonorae*	2019	Lanzarote	Arrecife	641223	3204522	3.18	3.74	0.41	0.45							3.89	2	4555	Pork
*Falco eleonorae*	2019	Lanzarote	Haria	645428	3238810	1.18	1.38									1.28	1	7345	Goat/sheep
*Falco eleonorae*	2016	Fuerteventura	Pájara	567311	3105914											0	0	19737	Pork
*Falco peregrinus pelegrinoides*	2020	Gran Canaria	Artenara	449358	3101476	1.10	1.30									1.2	1	430	Goat/Sheep
*Falco peregrinus pelegrinoides*	2019	Lanzarote	Teguise	634517	3215084	5.80	6.80									6.3	1	1343	Pork
*Falco peregrinus pelegrinoides*	2019	Gran Canaria	Telde	462858	3097310	7.27	8.53	7.10	7.70							15.3	2	1420	Goat/Sheep
*Falco peregrinus pelegrinoides*	2019	Gran Canaria	Telde	462858	3097310											0	0	1420	Goat/Sheep
*Falco peregrinus pelegrinoides*	2018	Tenerife	Arico	352926	3115789	3.54	4.16	4.77	5.17							8.82	2	1710	Cattle
*Falco peregrinus pelegrinoides*	2020	Gran Canaria	Agüimes	453150	3086742											0	0	3213	Goat/sheep
*Falco peregrinus pelegrinoides*	2019	Gran Canaria	Agüimes	459145	3081405											0	0	3655	Goat/Sheep
*Falco peregrinus pelegrinoides*	2011	Lanzarote	Tinajo	626292	3212418	29.37	23.07	39.12	33.32							62.44	2	4002	Pork
*Falco peregrinus pelegrinoides*	2011	Lanzarote	Yaiza	620344	3207276											0	0	4684	Goat/Sheep
*Falco peregrinus pelegrinoides*	2018	Gran Canaria	Mogan	438231	3089822											0	0	6213	Cattle
*Falco peregrinus pelegrinoides*	2011	Lanzarote	Teguise	640224	3215519	6.33	4.97									5.65	1	6816	Pork
*Falco peregrinus pelegrinoides*	2018	Gran Canaria	Mogan	437655	3088876											0	0	7432	Cattle
*Falco peregrinus pelegrinoides*	2015	Fuerteventura	La Oliva	615073	3180976											0	0	10343	Goat/Sheep
*Falco subbuteo*	2019	Fuerteventura	La Oliva	568210	3105010											0	0	20240	Pork
*Falco tinnunculus canariensis*	2019	Gran Canaria	Santa Brígida	456500	3101889	101.55	79.79	25.51	21.73							114.29	2	278	Pork
*Falco tinnunculus canariensis*	2020	Gran Canaria	Arucas	448651	3109655	109.87	86.33	61.34	52.26							154.9	2	288	Goat/Sheep
*Falco tinnunculus canariensis*	2020	Gran Canaria	San Bartolomé de Tirajana	443196	3087108	6.61	5.19	33.37	28.43	30.14	32.66			11.66	9.94	79	4	308	Cattle
*Falco tinnunculus canariensis*	2020	Gran Canaria	Ingenio	460439	3086554	14.09	11.07	4.60	3.92	25.14	27.24					43.03	3	380	Cattle
*Falco tinnunculus canariensis*	2020	Gran Canaria	Telde	461779	3097167	26.61	20.91	36.41	31.01	1.54	1.66			0.50	0.42	59.53	4	405	Goat/Sheep
*Falco tinnunculus canariensis*	2014	La Palma	Los Llanos de Aridane	216856	3173902	163.56	128.52	419.50	357.36	32.09	34.77					567.9	3	430	Pork
*Falco tinnunculus canariensis*	2014	Gran Canaria	Santa María de Guía	437007	3107606	97.52	76.62	560.20	477.20							605.77	2	492	Pork
*Falco tinnunculus canariensis*	2018	La Gomera	San Sebastian de la Gomera	293533	3109569	48.05	37.75			3.46	3.74					46.5	2	505	Pork
*Falco tinnunculus canariensis*	2014	La Palma	El Paso	224440	3161517											0	0	572	Goat/Sheep
*Falco tinnunculus canariensis*	2014	La Palma	El Paso	224440	3161517	28.13	35.09	1013.65	1076.35							1076.61	2	572	Goat/Sheep
*Falco tinnunculus canariensis*	2014	Gran Canaria	Valleseco	443613	3102482	47.56	59.32									53.44	1	695	Cattle
*Falco tinnunculus canariensis*	2020	Gran Canaria	Tejeda	444550	3092087	30.37	37.87	4.92	5.22	60.83	51.81	8.65	7.37			103.52	4	774	Cattle
*Falco tinnunculus canariensis*	2020	Gran Canaria	San Bartolomé de Tirajana	448717	3072935	22.70	28.31	26.00	27.60	1.94	1.66	2.59	2.21			56.5	4	820	Goat/Sheep
*Falco tinnunculus canariensis*	2020	Gran Canaria	Ingenio	456956	3088494	47.97	59.83	54.13	57.47	11.34	9.66					120.2	3	820	Goat/Sheep
*Falco tinnunculus canariensis*	2019	La Gomera	Hemigua	278774	3117473			14.74	15.66							15.2	1	894	Pork
*Falco tinnunculus canariensis*	2017	La Gomera	Alajeró	279727	3109537	605.20	754.80	50.44	53.56							732	2	909	Pork
*Falco tinnunculus canariensis*	2020	Gran Canaria	San Bartolomé de Tirajana	446416	3073529	5.74	7.16	1.12	1.18	1.19	1.01	2.91	2.47			11.39	4	911	Pork
*Falco tinnunculus canariensis*	2019	Gran Canaria	Las Palmas de Gran Canaria	457740	3100863	62.03	77.37	4.66	4.94							74.5	2	920	Cattle
*Falco tinnunculus canariensis*	2019	Gran Canaria	Arucas	447882	3112415	38.72	48.29	43.46	46.14	12.10	10.30					99.5	3	930	Goat/Sheep
*Falco tinnunculus canariensis*	2019	Gran Canaria	Santa Lucía de Tirajana	456584	3080365	15.31	19.09	18.33	19.47	1.51	1.29					37.5	3	950	Cattle
*Falco tinnunculus canariensis*	2020	Gran Canaria	San Bartolomé de Tirajana	440413	3073126	1.25	1.55	7.84	9.78	0.52	0.44	0.54	0.58			11.25	4	1023	Cattle
*Falco tinnunculus canariensis*	2020	Gran Canaria	San Bartolomé de Tirajana	441025	3072520	179.10	223.38	26.99	33.67	9.54	8.12					240.4	3	1120	Goat/Sheep
*Falco tinnunculus canariensis*	2014	La Palma	Los Llanos de Aridane	215137	3165875			1833.40	2286.60							2060	1	1123	Goat/Sheep
*Falco tinnunculus canariensis*	2014	La Palma	El Paso	219297	3172571			20.60	25.70							23.15	1	1266	Goat/Sheep
*Falco tinnunculus canariensis*	2014	La Palma	El Paso	224341	3162500			4307.60	5372.40							4840	1	1311	Pork
*Falco tinnunculus canariensis*	2020	Gran Canaria	Arucas	447238	3106246	56.37	66.17	3.85	4.81	0.46	0.40					66.03	3	1321	Cattle
*Falco tinnunculus canariensis*	2014	Gran Canaria	Firgas	443790	3109207	818.42	960.76	219.49	273.75							1136.21	2	1348	Goat/Sheep
*Falco tinnunculus canariensis*	2017	Tenerife	Los Realejos	343970	3142281											0	0	1352	Pork
*Falco tinnunculus canariensis*	2017	Tenerife	Tacoronte	362702	3148625			12.64	15.76							14.2	1	1385	Cattle
*Falco tinnunculus canariensis*	2018	Tenerife	La Orotava	351940	3140026	1.29	1.51			1.57	1.67					3.02	2	1423	Pork
*Falco tinnunculus canariensis*	2015	Gran Canaria	Las Palmas de Gran Canaria	452395	3110907	71.41	83.83	328.14	409.26							446.32	2	1430	Goat/Sheep
*Falco tinnunculus canariensis*	2018	Tenerife	Icod de los Vinos	332042	3136867	3.52	4.14	4.57	5.69							8.96	2	1463	Cattle
*Falco tinnunculus canariensis*	2016	Gran Canaria	Santa Brígida	453909	3102429	1079.69	1267.47	170.49	212.63							1365.14	2	1640	Goat/Sheep
*Falco tinnunculus canariensis*	2016	Gran Canaria	Santa Brígida	453976	3102516	909.15	1067.27									988.21	1	1640	Goat/Sheep
*Falco tinnunculus canariensis*	2017	La Gomera	Alajeró	279894	3110877	41.40	48.60	64.32	69.68							112	2	1718	Pork
*Falco tinnunculus canariensis*	2014	La Palma	Tazacorte	212224	3173709	11.93	14.01	75.64	81.94							91.76	2	1732	Goat/Sheep
*Falco tinnunculus canariensis*	2018	Tenerife	Garachico	329541	3139807	9.90	11.62	160.61	173.99	4.41	4.69					182.61	3	1788	Cattle
*Falco tinnunculus canariensis*	2018	Tenerife	El Tanque	325971	3138235	2.71	3.19	12.43	13.47							15.9	2	1836	Cattle
*Falco tinnunculus canariensis*	2018	Gran Canaria	Agaete	434493	3103475											0	0	1901	Goat/Sheep
*Falco tinnunculus canariensis*	2014	Gran Canaria	Telde	459249	3097315	11.92	14.00	281.54	305.00	23.90	25.38					330.87	3	1966	Goat/Sheep
*Falco tinnunculus canariensis*	2016	Gran Canaria	Galdar	438114	3103429			37.34	40.46							38.9	1	2167	Cattle
*Falco tinnunculus canariensis*	2017	Tenerife	Guía de Isora	324346	3123508	840.00	660.00	27.84	30.16							779	2	2332	Goat/Sheep
*Falco tinnunculus canariensis*	2017	Tenerife	Guía de Isora	324346	3123508	403.20	316.80	172.80	187.20							540	2	2332	Goat/Sheep
*Falco tinnunculus canariensis*	2018	La Gomera	San Sebastian de la Gomera	283452	3110317			581.76	630.24							606	1	2980	Pork
*Falco tinnunculus canariensis*	2017	Tenerife	Santiago del Teide	319264	3124971											0	0	3200	Goat/Sheep
*Falco tinnunculus canariensis*	2016	Gran Canaria	Las Palmas de Gran Canaria	458680	3104557	3.73	2.93	2.52	2.72							5.95	2	3601	Pork
*Falco tinnunculus canariensis*	2016	Gran Canaria	Las Palmas de Gran Canaria	458981	3106517											0	0	3601	Pork
*Falco tinnunculus canariensis*	2016	Gran Canaria	Las Palmas de Gran Canaria	458981	3106517											0	0	3601	Pork
*Falco tinnunculus canariensis*	2016	Gran Canaria	Las Palmas de Gran Canaria	458981	3106517											0	0	3601	Pork
*Falco tinnunculus canariensis*	2016	Gran Canaria	Las Palmas de Gran Canaria	458981	3106517											0	0	3601	Pork
*Falco tinnunculus canariensis*	2018	Tenerife	El Tanque	325000	3136221											0	0	3685	Cattle
*Falco tinnunculus canariensis*	2020	La Palma	Mazo	225396	3180471											0	0	3876	Goat/sheep
*Falco tinnunculus canariensis*	2020	Tenerife	El Tanque	337685	3137794											0	0	3965	Cattle
*Falco tinnunculus canariensis*	2020	Gran Canaria	Tejeda	444533	3071230											0	0	4380	Goat/Sheep
*Falco tinnunculus canariensis*	2017	Tenerife	Santa Cruz de Tenerife	323246	3127519	728.00	572.00	121.18	103.22							762.2	2	4460	Goat/Sheep
*Falco tinnunculus canariensis*	2017	Tenerife	Santa Cruz de Tenerife	323246	3127519	350.45	275.35	191.05	162.75							489.8	2	4460	Goat/Sheep
*Falco tinnunculus canariensis*	2017	Tenerife	El Tanque	324534	3134856											0	0	5070	Cattle
*Falco tinnunculus canariensis*	2017	Tenerife	El Tanque	324448	3134743											0	0	5160	Cattle
*Falco tinnunculus canariensis*	2014	Gran Canaria	San Mateo	444338	3095625	534.00	666.00									600	1	5279	Goat/Sheep
*Falco tinnunculus canariensis*	2020	Gran Canaria	San Bartolomé de Tirajana	441804	3069539											0	0	5432	Goat/sheep
*Falco tinnunculus canariensis*	2018	Tenerife	Buenavista del Norte	320372	3129173											0	0	6152	Goat/Sheep
*Falco tinnunculus canariensis*	2017	La Gomera	Hermigua	284055	3117368											0	0	6200	Goat/Sheep
*Falco tinnunculus canariensis*	2017	Tenerife	El Tanque	323743	3134459			10.04	8.56							9.3	1	6321	Pork
*Falco tinnunculus canariensis*	2018	Tenerife	Vilaflor	338727	3115304											0	0	6427	Cattle
*Falco tinnunculus canariensis*	2018	La Gomera	Alajeró	282492	3102551	34.43	42.93									38.68	1	6508	Pork
*Falco tinnunculus canariensis*	2018	Tenerife	Santiago del Teide	325564	3130959											0	0	7848	Cattle
*Falco tinnunculus canariensis*	2017	Tenerife	Santa Cruz de Tenerife	378838	3158973											0	0	10177	Pork
*Falco tinnunculus canariensis*	2017	Tenerife	Santa Cruz de Tenerife	378875	3158830											0	0	10177	Pork
*Falco tinnunculus dacotiae*	2019	Fuerteventura	Antigua	596424	3140592	24.83	30.97	31.64	26.96	2.04	2.16					59.3	3	497	Pork
*Falco tinnunculus dacotiae*	2019	Lanzarote	Haría	636900	3219658	190.10	237.10	49.46	42.14							259.4	2	1376	Pork
*Falco tinnunculus dacotiae*	2016	Lanzarote	Tinajo	629815	3214304			72.62	61.86							67.24	1	1493	Pork
*Falco tinnunculus dacotiae*	2019	Fuerteventura	La Oliva	608201	3172036											0	0	2312	Goat/Sheep
*Falco tinnunculus dacotiae*	2020	Fuerteventura	La Oliva	594156	3160227											0	0	2378	Cattle
*Falco tinnunculus dacotiae*	2020	Fuerteventura	Puerto del Rosario	604782	3153957											0	0	3156	Pork
*Falco tinnunculus dacotiae*	2019	Fuerteventura	Puerto del Rosario	609610	3152448	30.97	38.63	3.68	3.14	4.01	5.00					42.71	3	4104	Pork
*Falco tinnunculus dacotiae*	2011	Lanzarote	Tinajo	626294	3212311											0	0	4244	Pork
*Falco tinnunculus dacotiae*	2012	Lanzarote	Yaiza	613978	3208223											0	0	4480	Pork
*Falco tinnunculus dacotiae*	2019	Lanzarote	Teguise	640583	3216566											0	0	5617	Pork
*Falco tinnunculus dacotiae*	2020	La Gomera	Hemigua	287716	3114182											0	0	5789	Goat/sheep
*Falco tinnunculus dacotiae*	2017	Fuerteventura	Pájara	585926	3137560	11.13	13.88	3.15	3.35	1.07	1.33					16.95	3	8000	Goat/Sheep
*Falco tinnunculus dacotiae*	2020	Fuerteventura	Pájara	567931	3108609											0	0	8731	Goat/sheep
*Falco tinnunculus dacotiae*	2014	Fuerteventura	Pájara	581964	3137545	9.93	12.39									11.16	1	9275	Goat/Sheep
*Falco tinnunculus dacotiae*	2018	Lanzarote	Yaiza	613971	3208465											0	0	9596	Pork
*Neophron percnopterus majorensis*	2016	Fuerteventura	Tuineje	596547	3123216	124.57	146.23			11.06	13.80					147.83	2	204	Pork
*Neophron percnopterus majorensis*	2013	Gran Canaria	Las Palmas de Gran Canaria	455338	3104877											0	0	338	Goat/Sheep
*Neophron percnopterus majorensis*	2016	Fuerteventura	Tuineje	596547	3123216	21.30	25.00	74.91	79.55							100.38	2	455	Pork
*Neophron percnopterus majorensis*	2020	Fuerteventura	Pájara	560849	3108460	12.31	14.45	5.92	6.28							19.48	2	942	Pork
*Neophron percnopterus majorensis*	2017	Fuerteventura	Betancuria	590940	3143771	36.52	42.88									39.7	1	1080	Goat/Sheep
*Neophron percnopterus majorensis*	2016	Fuerteventura	Puerto del Rosario	607315	3150965			15.20	16.14							15.67	1	1125	Pork
*Neophron percnopterus majorensis*	2016	Fuerteventura	Puerto del Rosario	606445	3156687	26.59	31.21			2.38	2.96					31.57	2	1125	Pork
*Neophron percnopterus majorensis*	2016	Fuerteventura	Puerto del Rosario	607315	3150965											0	0	1125	Pork
*Neophron percnopterus majorensis*	2015	Fuerteventura	La Oliva	604823	3166247											0	0	1257	Goat/Sheep
*Neophron percnopterus majorensis*	2015	Fuerteventura	La Oliva	604823	3166247			41.97	44.57							43.27	1	1257	Goat/Sheep
*Neophron percnopterus majorensis*	2015	Fuerteventura	La Oliva	604823	3166247											0	0	1257	Goat/Sheep
*Neophron percnopterus majorensis*	2015	Fuerteventura	La Oliva	604823	3166247	30.77	36.13	28.03	29.77							62.35	2	1257	Goat/Sheep
*Neophron percnopterus majorensis*	2015	Fuerteventura	La Oliva	604823	3166247											0	0	1257	Goat/Sheep
*Neophron percnopterus majorensis*	2015	Fuerteventura	La Oliva	604823	3166247	70.42	82.66			18.79	23.43					97.65	2	1257	Goat/Sheep
*Neophron percnopterus majorensis*	2019	Fuerteventura	Pájara	607688	3159978	13.34	15.66	2.72	2.88							17.3	2	1294	Pork
*Neophron percnopterus majorensis*	2017	Fuerteventura	Antigua	598898	3143771	162.38	190.62									176.5	1	1320	Pork
*Neophron percnopterus majorensis*	2016	Fuerteventura	Puerto del Rosario	590936	3142556											0	0	1371	Goat/Sheep
*Neophron percnopterus majorensis*	2016	Fuerteventura	Puerto del Rosario	590936	3142556			36.69	45.77							41.23	1	1371	Goat/Sheep
*Neophron percnopterus majorensis*	2016	Fuerteventura	Antigua	605541	3130700											0	0	1397	Cattle
*Neophron percnopterus majorensis*	2016	Fuerteventura	Antigua	605541	3130700	29.77	37.13	100.08	124.82							145.9	2	1397	Cattle
*Neophron percnopterus majorensis*	2016	Fuerteventura	Puerto del Rosario	604312	3150277			28.59	35.65							32.12	1	1402	Pork
*Neophron percnopterus majorensis*	2016	Fuerteventura	Puerto del Rosario	611720	3155801											0	0	1434	Pork
*Neophron percnopterus majorensis*	2016	Fuerteventura	Puerto del Rosario	611720	3155801	11.77	14.69	29.56	36.86							46.44	2	1434	Pork
*Neophron percnopterus majorensis*	2017	Fuerteventura	Puerto del Rosario	607305	3150431			40.65	50.69							45.67	1	1514	Pork
*Neophron percnopterus majorensis*	2018	Fuerteventura	Puerto del Rosario	608263	3160015	8.28	10.32	88.91	110.89							109.2	2	1614	Pork
*Neophron percnopterus majorensis*	2017	Fuerteventura	Puerto del Rosario	596714	3153054											0	0	1703	Pork
*Neophron percnopterus majorensis*	2017	Fuerteventura	Puerto del Rosario	596714	3153054			11.76	14.66							13.21	1	1703	Pork
*Neophron percnopterus majorensis*	2018	Fuerteventura	Tuineje	596478	3134487											0	0	1822	Goat/Sheep
*Neophron percnopterus majorensis*	2017	Fuerteventura	Tuineje	587076	3127897											0	0	1850	Goat/Sheep
*Neophron percnopterus majorensis*	2018	Fuerteventura	Puerto del Rosario	606387	3150575	86.06	107.34									96.7	1	1997	Pork
*Neophron percnopterus majorensis*	2019	Fuerteventura	Puerto del Rosario	609021	3150578											0	0	2374	Pork
*Neophron percnopterus majorensis*	2016	Fuerteventura	La Oliva	612467	3167795											0	0	3266	Goat/Sheep
*Neophron percnopterus majorensis*	2012	Fuerteventura	La Oliva	604784	3171358											0	0	3758	Goat/Sheep
*Neophron percnopterus majorensis*	2015	Fuerteventura	La Oliva	604823	3166247											0	0	3787	Goat/Sheep
*Neophron percnopterus majorensis*	2015	Fuerteventura	La Oliva	604823	3166247											0	0	3787	Goat/Sheep
*Neophron percnopterus majorensis*	2015	Fuerteventura	La Oliva	604823	3166247											0	0	3787	Goat/Sheep
*Neophron percnopterus majorensis*	2015	Fuerteventura	La Oliva	604823	3166247											0	0	3787	Goat/Sheep
*Neophron percnopterus majorensis*	2019	Fuerteventura	Puerto del Rosario	607885	3144275											0	0	4320	Goat/Sheep
*Neophron percnopterus majorensis*	2017	Fuerteventura	Puerto del Rosario	611889	3160520											0	0	4320	Pork
*Neophron percnopterus majorensis*	2016	Fuerteventura	La Oliva	611994	3169859											0	0	4322	Goat/Sheep
*Neophron percnopterus majorensis*	2016	Fuerteventura	La Oliva	599608	3168201											0	0	5137	Goat/Sheep
*Neophron percnopterus majorensis*	2017	Fuerteventura	Puerto del Rosario	609614	3144261											0	0	5311	Pork
*Neophron percnopterus majorensis*	2017	Fuerteventura	Tuineje	593053	3140264											0	0	5438	Goat/Sheep
*Neophron percnopterus majorensis*	2017	Fuerteventura	Antigua	608244	3142737											0	0	5783	Goat/Sheep
*Neophron percnopterus majorensis*	2017	Fuerteventura	Pájara	587946	3134649											0	0	5890	Goat/Sheep
*Neophron percnopterus majorensis*	2019	Lanzarote	Teguise	645605	3218612											0	0	6023	Goat/Sheep
*Neophron percnopterus majorensis*	2017	Fuerteventura	La Oliva	598129	3168975											0	0	6214	Pork
*Neophron percnopterus majorensis*	2019	Fuerteventura	La Oliva	597873	3166603											0	0	6251	Goat/Sheep
*Neophron percnopterus majorensis*	2017	Fuerteventura	Puerto del Rosario	605347	3134467											0	0	6450	Goat/Sheep
*Neophron percnopterus majorensis*	2017	Fuerteventura	Pájara	585159	3141910											0	0	6580	Goat/Sheep
*Neophron percnopterus majorensis*	2019	Fuerteventura	Puerto del Rosario	604194	3130953											0	0	6790	Goat/Sheep
*Neophron percnopterus majorensis*	2017	Fuerteventura	Tuineje	608844	3137625											0	0	7125	Goat/Sheep
*Neophron percnopterus majorensis*	2017	Fuerteventura	Pájara	583630	3134807											0	0	7230	Goat/Sheep
*Neophron percnopterus majorensis*	2018	Fuerteventura	Pájara	573786	3115739											0	0	7719	Pork
*Neophron percnopterus majorensis*	2015	Fuerteventura	La Oliva	604823	3166247											0	0	7750	Goat/Sheep
*Neophron percnopterus majorensis*	2015	Fuerteventura	La Oliva	604823	3166247											0	0	7750	Goat/Sheep
*Neophron percnopterus majorensis*	2020	Fuerteventura	Pájara	560849	3109790											0	0	8201	Goat/Sheep
*Neophron percnopterus majorensis*	2020	Fuerteventura	Pájara	561500	3108762											0	0	8201	Goat/Sheep
*Neophron percnopterus majorensis*	2020	Fuerteventura	Pájara	560847	3108070											0	0	8201	Goat/Sheep
*Neophron percnopterus majorensis*	2020	Fuerteventura	Pájara	562108	3108323											0	0	8201	Goat/Sheep
*Neophron percnopterus majorensis*	2020	Fuerteventura	Pájara	561541	3108920											0	0	8201	Goat/Sheep
*Neophron percnopterus majorensis*	2020	Fuerteventura	Pájara	562037	3108820											0	0	8201	Goat/Sheep
*Neophron percnopterus majorensis*	2011	Fuerteventura	Tuineje	590891	3124411	12.21	9.59	20.29	21.99							32.04	2	8240	Pork
*Neophron percnopterus majorensis*	2020	Fuerteventura	Pájara	582566	311811											0	0	8544	Goat/Sheep
*Neophron percnopterus majorensis*	2020	Fuerteventura	Puerto del Rosario	603683	3153678											0	0	9716	Goat/sheep
*Neophron percnopterus majorensis*	2015	Fuerteventura	Pájara	581745	3137832											0	0	9870	Goat/Sheep
*Neophron percnopterus majorensis*	2017	Fuerteventura	Pájara	562439	3109174											0	0	20147	Pork
*Tyto alba alba*	2020	Gran Canaria	Galdar	438947	3108983	14.29	11.23	3.08	3.34	1.11	1.39					17.22	3	811	Pork
*Tyto alba alba*	2019	Gran Canaria	Galdar	437861	3109172	5.60	4.40	1.54	1.66	13.88	17.32					22.2	3	867	Cattle
*Tyto alba alba*	2020	Gran Canaria	Galdar	432671	3116177	134.06	105.34									119.7	1	1104	Pork
*Tyto alba alba*	2020	Gran Canaria	Santa Brígida	451999	3100544											0	0	1480	Goat/Sheep
*Tyto alba alba*	2019	Gran Canaria	Las Palmas de Gran Canaria	450683	3107583	4.82	3.78									4.3	1	3325	Goat/Sheep
*Tyto alba gracilirostris*	2011	Lanzarote	Yaiza	623148	3198152	0.20	0.16	0.22	0.24	0.04	0.04					0.45	3	602	Pork
*Tyto alba gracilirostris*	2019	Fuerteventura	Puerto del Rosario	597146	3152290	12.21	9.59									10.9	1	1400	Pork
*Tyto alba gracilirostris*	2011	Lanzarote	Yaiza	619010	3203526	12.77	10.03	32.06	34.74			1.13	1.19			45.96	3	1423	Pork

**Fig. 1:**
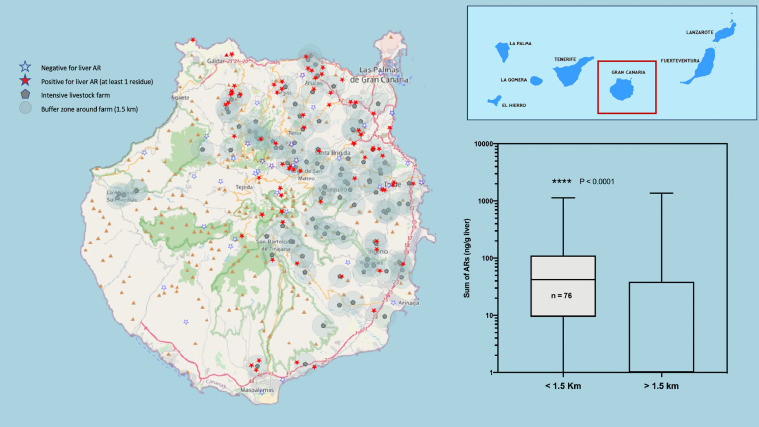
Map of the Island of Gran Canaria. The location of the cases of raptors positive to oral anticoagulants (red stars), the negative ones (white stars) and the cattle farms surrounded by a buffer zone of 1.5 km radius are shown. On the right, a box and whiskers graph shows the statistical comparison between the two groups of animals, found inside or outside the buffer zone of the farms. (For interpretation of the references to color in this figure legend, the reader is referred to the web version of this article.)

**Fig. 2:**
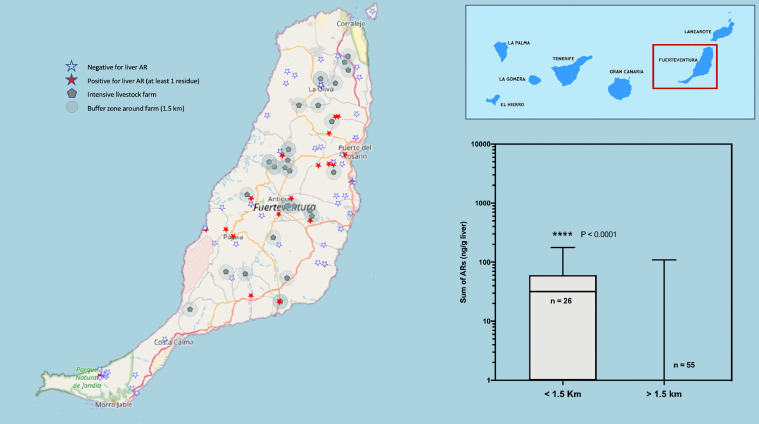
Map of the Island of Fuerteventura. The location of the cases of raptors positive to oral anticoagulants (red stars), the negative ones (white stars) and the cattle farms surrounded by a buffer zone of 1.5 km radius are shown. On the right, a box and whiskers graph shows the statistical comparison between the two groups of animals, found inside or outside the buffer zone of the farms. (For interpretation of the references to color in this figure legend, the reader is referred to the web version of this article.)

**Fig. 3:**
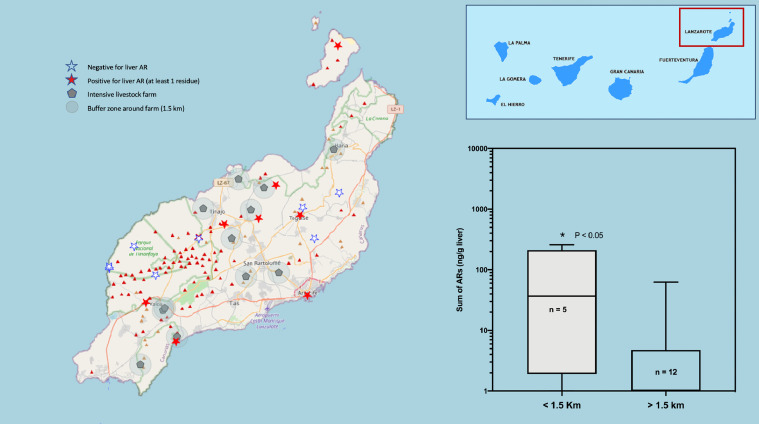
Map of the Islands of Lanzarote and La Graciosa. The location of the cases of raptors positive to oral anticoagulants (red stars), the negative ones (white stars) and the cattle farms surrounded by a buffer zone of 1.5 km radius are shown. On the right, a box and whiskers graph shows the statistical comparison between the two groups of animals, found inside or outside the buffer zone of the farms. (For interpretation of the references to color in this figure legend, the reader is referred to the web version of this article.)

**Fig. 4:**
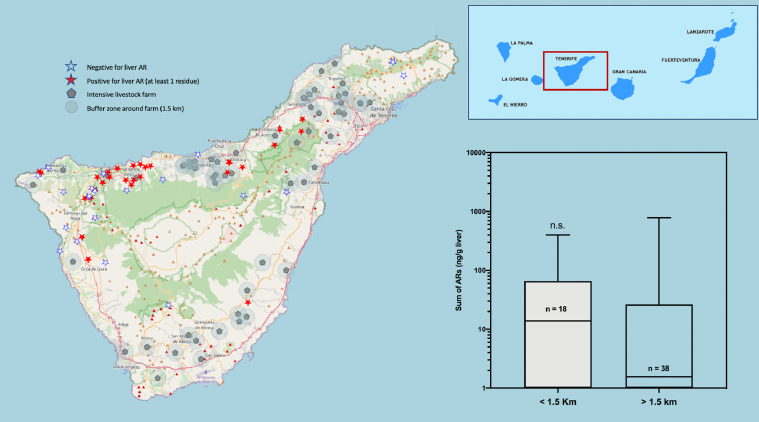
Map of the Island of Tenerife. The location of the cases of raptors positive to oral anticoagulants (red stars), the negative ones (white stars) and the cattle farms surrounded by a buffer zone of 1.5 km radius are shown. On the right, a box and whiskers graph shows the statistical comparison between the two groups of animals, found inside or outside the buffer zone of the farms. (For interpretation of the references to color in this figure legend, the reader is referred to the web version of this article.)

**Fig. 5:**
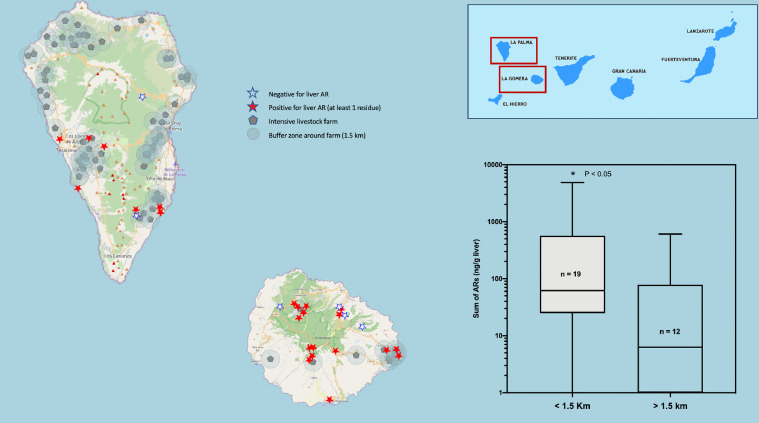
Map of the Islands of La Palma and La Gomera. The location of the cases of raptors positive to oral anticoagulants (red stars), the negative ones (white stars) and the cattle farms surrounded by a buffer zone of 1.5 km radius are shown. On the right, a box and whiskers graph shows the statistical comparison between the two groups of animals, found inside or outside the buffer zone of the farms. (For interpretation of the references to color in this figure legend, the reader is referred to the web version of this article.)

Figs. 1 to 5 present a double panel each. On the one hand, we present the geographical location of all cases (positive and negative for anticoagulant rodenticides) on the map of each of the Canary Islands (with the exception of El Hierro, in which only 2 cases were recorded), and the relationship with the location of medium / large-sized livestock farms, surrounded by a 1.5 km buffer zone (which we consider to coincide with the average home range of the raptor species studied). Additionally, in each of these graphs, the comparative statistics of the distribution of anticoagulant rodenticide values between the groups of animals that were found inside or outside the aforementioned 1.5 km buffer zone of the livestock farms are also presented.

**Fig. 6:**
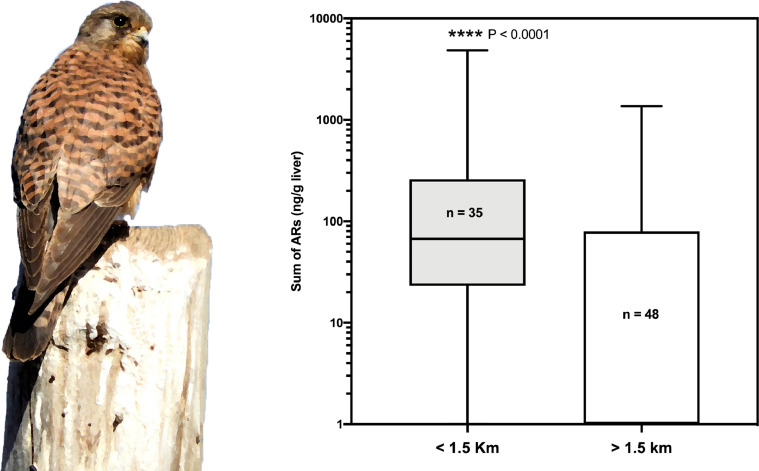
Box and whiskers graph showing the statistical comparison between the two groups of common buzzards (*Buteo buteo insularum*), found inside or outside the buffer zone of the farms.

**Fig. 7:**
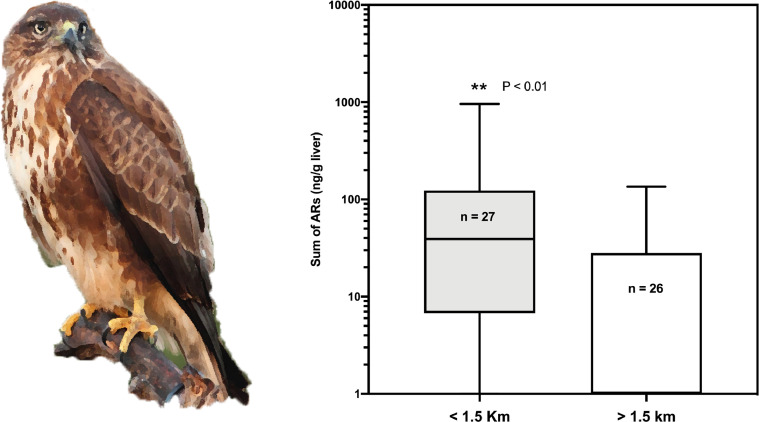
Box and whiskers graph showing the statistical comparison between the two groups of common kestrels (*Falco tinnunculus canariensis* and *dacotiae*), found inside or outside the buffer zone of the farms.

**Fig. 8:**
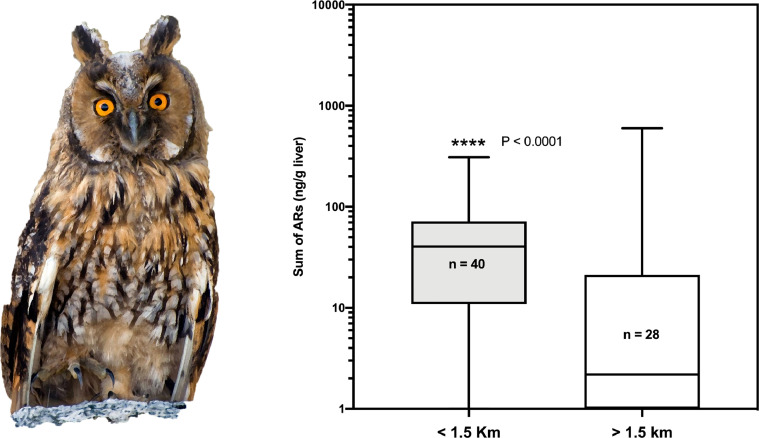
Box and whiskers graph showing the statistical comparison between the two groups of long-eared owls (*Asio otus canariensis*), found inside or outside the buffer zone of the farms.

**Fig. 9:**
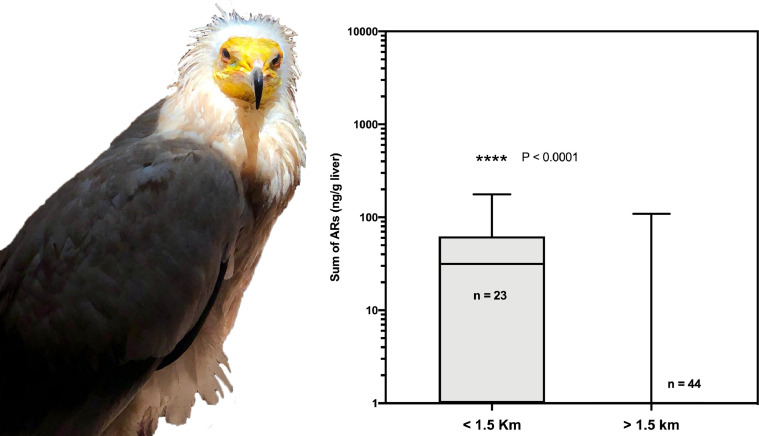
Box and whiskers graph showing the statistical comparison between the two groups of Egyptian vultures (*Neophron percnopterus majorensis*), found inside or outside the buffer zone of the farms.

Additionally, detailed analysis is presented for those raptor species in which more than 50 individuals were analysed. In these analyses we present the comparative statistics of the distribution of anticoagulant rodenticide values between the groups of birds that were found inside or outside the 1.5 km-buffer zone of farms: common kestrel (*Falco tinnunculus,* Fig. 6); common buzzard (*Buteo buteo*, Fig. 7); long-eared owl (*Asio otus*, Fig. 8); and Egyptian vulture (*Neophron percnopterus,* Fig. 9).

## 2 Experimental Design, Materials and Methods

### 2.1 Sampling

This study was carried out in the Canary Islands, and the samples were taken in the context of the Poisoning Control and Prevention Strategy in the Canaries [2] from 2011 to May 2020. The corpses or liver samples were received in the ULPGC Toxicology Laboratory for forensic toxicological evaluations. Only those birds that had georeferenced information about the place where they were found and furthermore, the good state of conservation of the animals allowed the sampling of the liver were included. The series of raptors included 308 individuals from 13 different species/subspecies: *Accipiter nisus granti* (*n* = 9); *Actitis hypoleucos* (*n* = 1); *Asio otus canariensis* (*n* = 68); *Buteo buteo insularum* (*n* = 53); *Circus aeruginosus* (n = 1); *Falco eleonorae* (n = 4); *Falco peregrinus pelegrinoides* (n = 13); *Falco subbuteo* (n = 1); *Falco tinnunculus canariensis* (*n* = 69); *Falco tinnunculus dacotiae* (n = 14); *Neophron percnopterus majorensis* (n = 67); *Tyto alba alba* (n = 5); and *Tyto alba gracilirostris* (*n* = 3). The animals were sent by environmental officers or patrols if found dead, or by wildlife recovery centers if they had been admitted alive but euthanized or death within a week of admission. All carcasses were kept frozen at −20 °C, until they were necropsied. No animals were sacrificed for the purpose of this study. The livers, as the main organ for accumulation and storage of rodenticides, were used for this study [3]. Obtained during the necropsy, they were kept frozen at −20 °C until the preparation of the extraction and chemical analysis.

### 2.2 Chemical analyses

During these 10 years, we employed two extraction-detection methods for the quantitative analysis of all the anticoagulant rodenticides permitted in the EU (brodifacoum, bromadiolone, chlorophacinone, coumatetralyl, difenacoum, difethialone, flocoumafen and warfarin) [4]. All the solvents employed were of the highest purity available (>99. 9%, Honeywell, Morristown, NJ, USA). Ultrapure (UP) water was produced in the laboratory using a Gradient A10 Milli-Q System (Millipore, Molsheim, France). Standards for ARs and a procedural-internal standard (P-IS, (±)-Warfarin-d5) were purchased from Dr. Ehrenstorfer (Augsburg, Germany). All standards were pure compounds (purity from 98% to 99.5%). The method employed from January 2011 to November 2015 was a solid-liquid extraction followed of a LC-MS/MS analysis using a Thermo LC-MS/MS Accela Ultra instrument (Thermo Fisher Scientific Inc., USA) as previously described [5]. The method employed from December 2015 to May 2020 consisted on an extraction based on the QuEChERS method (Anastassiades et al., 2003), which has been fully validated in our laboratory followed by a LC-MS/MS analysis using an Agilent 1290 UHPLC (Agilent Technologies, Palo Alto, USA) coupled to an Agilent 6460 triple-quadrupole mass spectrometer, according to the previously described procedure [6]. All the quantitative data were obtained from at least two independent measurements.

### 2.3 Geospatial analysis of the data (GIS analysis)

The data about the place where the carcass was found were collected by Canary Islands environmental patrols and obtained by GPS tracking. We employed the QGIS Desktop software (version 3.12) for the analyses of geospatial data. The images were projected to the UTM 28N zone based on the WGS84 Geographic Coordinates System. Several vectorial map layers were created for animals positive for anticoagulant rodenticides; animals negative for anticoagulant rodenticides, farms of pig/cattle/sheep/goat production, and 1.5 km-buffer zones for farms, and all these were superimposed on the base map (OpenStreetMap).

### 2.4 Statistical analysis

The statistical analyses were done using the software package using GraphPad Prism v8.0 (GraphPad Software, CA, USA). First, the adjustment to the series of data to normality was examined using the Kolmogorov–Smirnov test. The distributions of the anticoagulant rodenticides did not adjust to normality and therefore, non-parametric tests were employed. Thus, we used the Mann–Whitney tests for the analysis. Probability levels of less than 0.05 (two tailed) were considered statistically significant.

## Ethics Statement

All samples were collected after obtaining the corresponding permits and following the animal welfare protocols during the sampling.

## Declaration of Competing Interest

The authors declare that they have no known competing financial interests or personal relationships which have, or could be perceived to have, influenced the work reported in this article.
